# Current challenges and recent advances in the search for a cure for HIV

**DOI:** 10.1002/jia2.25248

**Published:** 2019-02-07

**Authors:** R Brad Jones

**Affiliations:** ^1^ Infectious Diseases Division Weill Cornell Medicine New York NY USA; ^2^ Department of Microbiology Immunology and Tropical Medicine The George Washington University Washington DC USA

**Keywords:** HIV cure, immunology, ARV, virology, clonal expansion, HIV reservoir

1

Although modern antiretroviral (ARV) therapies durably suppress HIV replication to undetectable levels, they are unable to cure infection. If therapy is ever interrupted, virus rapidly rebounds from persistent reservoirs of cells infected with HIV. A fundamental goal of HIV cure research is thus to develop novel therapies capable of targeting and eliminating viral reservoirs, which may allow individuals to stop ARV therapy without viral rebound. Here, we present a perspective on two key areas of challenge and advancement in relation to this goal.

## 2 Challenges and advances in measuring the HIV reservoir

The rational development and evaluation of interventions designed to reduce HIV reservoirs are currently impaired by limitations in our ability to measure these reservoirs both on a cellular level, and throughout the body. As an example of a specific challenge that has benefited from recent advances, some 98% of the HIV DNA as measured by conventional quantitative PCR methods represents defective proviruses that is viral genomes which are inactivated by large deletions or other mutations and thus have no ability to give rise viral rebound 1, 2, 3, 4. In response to this striking observation, a number of novel assays have been developed with the ability to distinguish intact versus defective proviruses. These assays range from near full‐length sequencing approaches, which give a full picture of the genomic make‐up of individual proviruses 1, 2, 3, 4, to a novel droplet digital PCR‐based “intact proviral DNA assay (IPDA),” which provides less information on individual viral genomes but offers rapid results and scalability 5.

**Figure 1:**
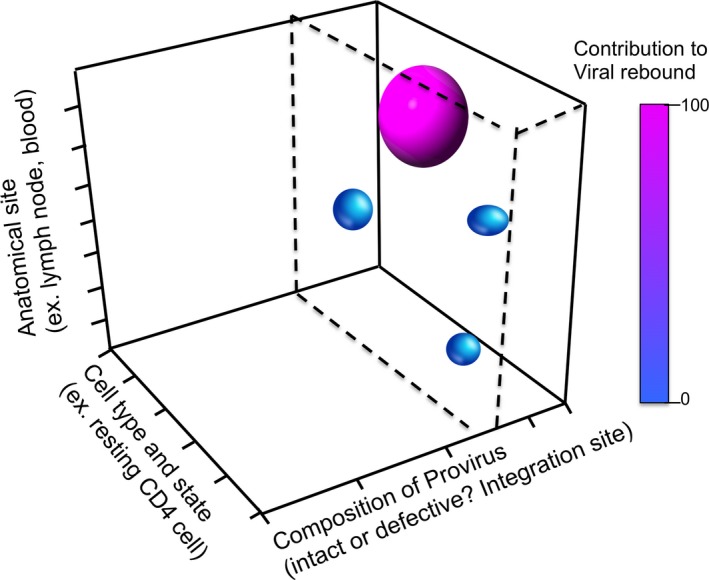
Hypothetical depiction of the landscape of cells infected with HIV in an individual, and contribution to viral rebound. The body‐wide landscape of infected is shown conceptually defined across three axes: (1) the anatomical site in which an infected cell is located (e.g. lymph node), (2) the type and state of that infected cell (e.g. resting CD4 cell), and (3) the composition of the HIV provirus in that infected cell (e.g. defective due to a large deletion). The shaded globes represent populations of infected cells that give rise to viral rebound on ART cessation. For example, the pink globe, which is contributing substantially, may correspond to resting CD4 cells in lymph nodes infected with replication competent virus. The dashes lines represent the improved ability to define the infected cells that contribute to rebound achieved by assays which distinguish defective from intact proviruses, where only the latter (to the right of the dashed lines) have the potential to contribute to viral rebound.

To contextualize the utility of these novel assays with respect to the broader reservoir, we propose that the landscape of the body‐wide cells infected with HIV can hypothetically by plotted against the following three dimensions: (1) anatomical site (e.g. lymph node, blood); (2) cell type and state (e.g. resting CD4 cell, macrophage); and (3) composition of HIV proviruses (intact or defective). As is depicted in Figure 1, we anticipate that within this three‐dimensional space there would be nodes that disproportionately contribute to viral rebound and other spaces that have no contributions. One hypothetical example of the former may be resting CD4^+^ T cells harbouring intact proviruses in lymph nodes, whereas perhaps defective proviruses in astrocytes in the brain may represent the latter.

The novel assays described above, which distinguish intact from defective proviruses, substantially improve our ability to limit the “Composition of Provirus” dimension to the proviruses with the potential to cause viral rebound (as indicated by the dashed lines). There is an ongoing need for basic and translational science to continue to develop our understanding of how the HIV reservoir is distributed *in vivo* with implications both for the appropriate targeting of interventions and for the measurement of any resulting perturbations.

## 3 Advances in understanding clonal expansion of HIV reservoirs and possible implications

Since the early 2000s, it has been known that the replication‐competent HIV reservoir is remarkably stable, with a decay half‐life of approximately 43 months 6, 7. Until recently, this stability was generally thought to reflect a static reservoir – whereby a pool of long‐lived infected resting CD4^+^ T cells simply persisted in a quiescent state.

What we have learned recently is that cells infected with HIV actually divide and proliferate in people living with HIV, a process known as clonal expansion 8, 9, 10, 11. This initially seemed at odds with the stable total frequency of infected cells, and implied that some infected cells must be dying off naturally over time, in order to reconcile these observations. Recent studies have demonstrated this explicitly, by looking at multiple different clones of cells infected with HIV, and seeing that the frequencies of these “waxed and waned” over time 12, 13.

A case can be made for the possibility that these newly appreciated dynamics open the door for HIV reservoirs to undergo Darwinian evolution within people living with HIV, considering the infected cells themselves (rather than virus) as the biological units. The requirements for evolution to occur over time are: (1) variation in a population; (2) replication and heritability; and (3) selective pressure 14. It is clear that there will be variation within a population of infected cells – contributed by natural heterogeneity among CD4^+^ T cells, by viral factors (e.g. intact vs. defective provirus), and perhaps, by changes arising from the proviral integration site 15, 16. Clonal expansion now satisfies the second requirement, by allowing cells to increase in numbers and pass on their characteristics to progeny. Several forces *in vivo* have the potential to apply selective pressure to infected cells, perhaps most notably immune pressure such as that applied by cytotoxic T cells (CTL) (immune cells that kill virus infected cells). We propose that if infected cells differ in their intrinsic susceptibility to CTL, this may result in a reservoir that has been selected for cells that were CTL resistant. This could potentially underlie our recent study which reported that “Latent HIV reservoirs exhibit inherent resistance to elimination by CD8^+^ T cells” 17 as well as the work of others showing overexpression of prosurvival factors in reservoir‐harbouring cells 18, 19.

The question of whether or not long‐lived reservoir‐harbouring cells have been selected by evolutionary processes may be one of the more critical questions being asked by the field, with potentially profound implications for efforts to eliminate these cells. Some potential approaches to address this involve: (1) Studying changes in the reservoir landscapes that occur over time in individuals living with HIV following ARV initiation in relation to potential drivers of selection, and (2) additional functional characterization of *ex vivo* reservoir‐harbouring cells, which would be enabled by novel approaches to isolating these rare populations. The identification of novel host‐cell characteristics that support HIV persistence may offer novel therapeutic targets that could be exploited along with latency reversal to reduce HIV reservoirs and bring us closer to a cure for HIV.

## Competing interests

RBJ declares that he is a member of the scientific advisory board of AbbVie Inc, and that he has no other potential conflicts of interest.
